# HIV-1 Envelope Glycoprotein at the Interface of Host Restriction and Virus Evasion

**DOI:** 10.3390/v11040311

**Published:** 2019-03-30

**Authors:** Saina Beitari, Yimeng Wang, Shan-Lu Liu, Chen Liang

**Affiliations:** 1 Department of Microbiology & Immunology, McGill University, Montreal, QC H3A 2B4, Canada; saina.beitari@mail.mcgill.ca; 2 Lady Davis Institute, Jewish General Hospital, Montreal, QC H3T 1E2, Canada; yimeng.wang@mail.mcgill.ca; 3 Department of Medicine, McGill University, Montreal, QC H4A 3J1, Canada; 4 Center for Retrovirus Research, Department of Veterinary Biosciences, Department of Microbial Infection and Immunity, The Ohio State University, Columbus, Ohio 43210, USA; liu.6244@osu.edu

**Keywords:** viral envelope protein, HIV, host restriction factor, viral antagonism, viral entry, innate immunity, adaptive immunity

## Abstract

Without viral envelope proteins, viruses cannot enter cells to start infection. As the major viral proteins present on the surface of virions, viral envelope proteins are a prominent target of the host immune system in preventing and ultimately eliminating viral infection. In addition to the well-appreciated adaptive immunity that produces envelope protein-specific antibodies and T cell responses, recent studies have begun to unveil a rich layer of host innate immune mechanisms restricting viral entry. This review focuses on the exciting progress that has been made in this new direction of research, by discussing various known examples of host restriction of viral entry, and diverse viral countering strategies, in particular, the emerging role of viral envelope proteins in evading host innate immune suppression. We will also highlight the effective cooperation between innate and adaptive immunity to achieve the synergistic control of viral infection by targeting viral envelope protein and checking viral escape. Given that many of the related findings were made with HIV-1, we will use HIV-1 as the model virus to illustrate the basic principles and molecular mechanisms on host restriction targeting HIV-1 envelope protein.

## 1. Introduction

The HIV-1 envelope (Env) protein is synthesized as a gp160 precursor at the endoplasmic reticulum (ER). Following complex glycosylation and cleavage by furin proteases at the trans-Golgi complex, the mature gp120/gp41 trimer travels to the plasma membrane where it joins HIV-1 Gag proteins, forming infectious virus particles (reviewed in [1]). To start a new round of infection, gp120 binds to the CD4 receptor on the surface of the target cell, which triggers conformational changes of the gp120/gp41 trimer, exposing the binding site in gp120 for the CCR5 or the CXCR4 co-receptor. Engagement of the co-receptor leads to the exposure of the fusion peptide followed by the assembly of the 6-helix bundle in gp41, causing the fusion of viral and cellular membranes. Membrane fusion begins with the joining of both membranes’ outer lipid leaflets in a process called hemifusion. Continued fusion of the two membranes leads to the formation of the fusion pore. The fusion pore further dilates to an adequate size for the delivery of HIV-1 RNA within the core structure into the cytoplasm.

As the only viral protein on the surface of HIV-1 particles, the Env protein represents the chief target for recognition by the host adaptive immune system, leading to the production of antibodies that recognize and bind to Env protein. Despite the various strategies that HIV-1 has exploited to evade neutralizing antibodies, including heavy glycosylation to mask the epitopes [2], a series of antibodies that are able to neutralize diverse HIV-1 strains has been isolated from HIV patients, called broadly neutralizing antibodies (bnAbs) (reviewed in [3]). The discovery of bnAbs has fueled HIV vaccine research through the characterization of bnAb epitopes on the Env protein and the elucidation of the B cell generation of bnAbs [4].

In addition to neutralizing HIV-1 particles, some Env-targeting antibodies are able to recruit natural killer (NK) cells through engaging the FC receptor, and kill HIV-1 infected cells by antibody-dependent cell-mediated cytotoxicity (ADCC) [5]. The ADCC-mediating Env Abs were isolated from subjects enrolled in the RV144 vaccine trial, which has shown a modest 31.2% protection efficacy [6]. Not surprisingly, HIV-1 has mechanisms to evade ADCC, including the use of accessory proteins Vpu and Nef to downregulate CD4, which otherwise interacts with the Env protein to expose epitopes of ADCC-triggering antibodies (reviewed in [7]).

## 2. HIV-1 Env Is Attacked by Host Innate Immunity

In addition to the constitutively active intrinsic innate immune mechanisms, the main stream of innate immunity is elicited upon host recognition of pathogen-associated molecular patterns (PAMPs) by a group of host proteins that act as pathogen recognition receptors (PRRs). This recognition event triggers signaling cascades that lead to the expression of cytokines, including interferons (IFNs). One function of these cytokines is to induce the expression of proteins that can directly restrict viral infections. The other equally important function of cytokines is to activate immune cells and initiate the pathogen-specific adaptive immune response.

By acting together, the constitutively expressed and interferon-induced antiviral proteins form the first line of host defense against viral infections. These antiviral proteins operate by a variety of molecular mechanisms to target distinct steps in the virus life cycle. As viral nucleic acids are the main PAMPs to induce innate immune response, they have also become the target of many cellular antiviral factors (reviewed in [8]). Examples include (1) nucleases, such as ISG20, OAS/RNase L, and three primer repair exonuclease 1 (TREX1) that degrade viral RNA or DNA; (2) deaminases, such as adenosine deaminases acting on RNA (ADAR) and apoliprotein B editing complex 3 (APOBEC3) proteins that edit and mutate the viral genome; (3) dNTP hydrolase SAM domain and HD domain-containing protein 1 (SAMHD1), which diminishes the cellular DNA pool and inhibits viral DNA synthesis; (4) factors, such as Myxovirus resistance 1, 2 (Mx1, Mx2), and Tripartite motif-containing protein 5α (Trim5α) that target the replication complex of viral genome and block viral multiplication; and (5) factors such as schlafen 11 (SLFN11) and protein kinase activated by RNA (PKR), which inhibit the translation of viral RNA [9,10,11,12,13,14,15,16].

In addition to these diverse cellular mechanisms that attack viral nucleic acids, recent studies have discovered an array of cellular proteins that contribute to the control of viral infection by targeting viral Env and inhibiting viral entry, which began to unravel a new layer of host antiviral defense. Through these studies, we have come to appreciate the combinatorial strategy that cells have evolved to restrict viral entry by targeting virtually every stage of Env’s life, from Env synthesis and maturation, to its incorporation into virus particles, to its execution of membrane fusion.

## 3. A Long and Challenging Journey for Env Protein to Reach Virus Particles

**Figure 1:**
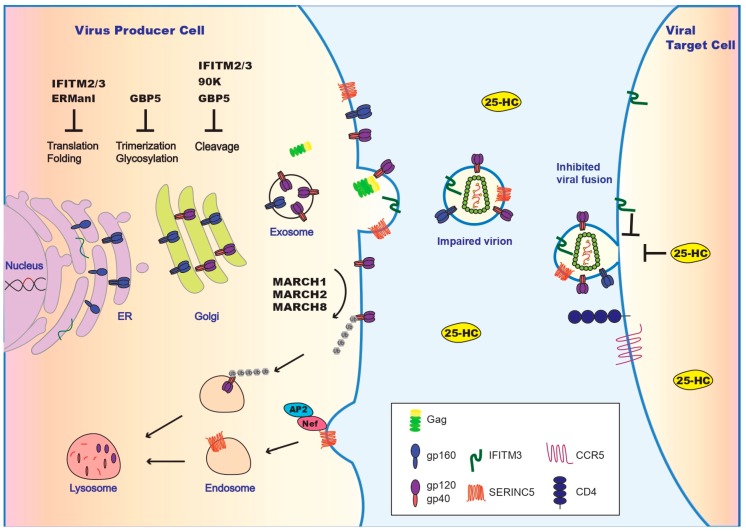
Inhibition of HIV-1 entry by restriction factors and viral counter measures. Illustrated are restriction factors that operate in virus producer cells and inhibit Env synthesis at the endoplasmic reticulum (ER) (by IFITM2/IFITM3 and ERManl), impair Env maturation at Golgi (by IFITM2/IFITM3, GBP5, and 90K), and downregulate Env at the plasma membrane (by MARCH1/MRCH2/MARCH8). IFITM2/IFITM3 and SERINC5 get incorporated into HIV-1 particles and impair viral membrane fusion. In virus target cells, IFITM2/IFITM3 and 25-HC deter viral entry. HIV-1 uses Nef to downregulate SERINC5. The other viral countering strategies are summarized in Table 1. A more comprehensive illustration of antiviral restriction factors is presented in [19].

From its de novo synthesis at the ER, HIV-1 Env protein travels through the trans-Golgi complex, arriving at the plasma membrane to join HIV-1 Gag proteins, together forming infectious progeny virions. Env can choose to detour to endosome recycle compartments (ERC), where it reaches the particle assembly site on the plasma membrane by interacting with FIP1C and Rab14 [17,18]. Along this journey, Env undergoes complex glycosylation, trimerization, and cleavage by furin, providing ample opportunities for the host cell to attack (Figure 1).

### 3.1. ER-Associated Degradation: Traps at Its Place of Birth

The newly synthesized Env precursor, gp160, folds at the ER with low efficiency [20]. The often misfolded Env protein is subjected to ER-associated degradation (ERAD), a process that controls and eliminates misfolded proteins [21]. One ER protein, called ERManI, has been shown to promote degradation of ER-associated proteins by ERAD. ERManI is a class I α-mannosidase and belongs to the glycosidehydrolase family 47 (GH47) α-mannosidases, which are carbohydrate-active enzymes [22]. GH47 α-mannosidases mediate the trimming of α-1,2-mannose residues from Man9GlcNAc2, among which ERManI is the first enzyme to generate Man8GlcNAc2 [23]. ERManl interacts with HIV-1 Env via its luminal catalytic domain, and mutation of the catalytic sites ablates its activity in degrading HIV-1 Env protein [24]. This function of ERManl is specific, because overexpression of other α-mannosidases from the family of GH47 α-mannosidases, such as ER-degradation enhancing α-mannosidase-like (EDEM) proteins 1, 2, and 3, does not affect HIV-1 Env expression [24]. It is thus speculated that ERManI operates by modulating glycosylation of HIV-1 Env. Interestingly, ERManI is required for the function of a mitochondrial translocator protein called TSPO in diminishing HIV-1 Env expression via ERAD since ERManI depletion abolished TSPO-mediated Env degradation [24,25].

The antiviral activity of ERManI is not limited to the degradation of HIV-1 Env protein, and the hemagglutinin (HA) glycoprotein of influenza virus is also a target of ERManI and the ERAD pathway [26]. To date, viral countermeasures of ERManI have not been reported [24]. However, HIV-1 Vpr has been shown to increase Env expression [27]. In the absence of Vpr, Env tends to misfold and is targeted to the ERAD pathway for degradation. The N-terminal region of Vpr controls this activity, since a single A30L mutation disrupts the function of Vpr to increase Env expression [27]. It remains to be determined whether Vpr directly interacts with ERManI to save Env from degradation at the ER.

The ERAD-mediated degradation of HIV-1 Env can be exploited for therapeutic purposes. One example is the depletion of HIV-1 gp160 precursor from the ER via the ERAD pathway by an engineered molecule called degradin, which contains gp120-targeting antibody chains and the C-terminal sequence of ER-resident protein SEL1L [28]. By a similar mechanism, the small peptide glycine-prolyl-glycine amide (GPG-NH2) abolishes HIV-1 infectivity by targeting viral Env to the ERAD pathway for degradation [29]. The ERAD pathway appears to be a double-edged sword, since HIV-1 Vpu hijacks this protein degradation mechanism to remove CD4 from the ER [30]. Premature contact of Env with CD4 is thus avoided to ensure that Env is safely transported to the plasma membrane for virus assembly.

### 3.2. GBP5, 90K, and IFITM3: Blocks Along Env’s Route to the Virus Assembly Site

The precursor of HIV-1 Env, gp160, trimerizes at the ER then moves to the trans-Golgi apparatus where it is cleaved to become gp120/gp41, forming a mature Env trimer [1]. Products of three interferon-stimulated genes (ISGs), GBP5 (guanylate binding protein 5), 90K, and IFITM2/3 (IFN-induced transmembrane protein 2 and 3), have been shown to obstruct gp160 cleavage and diminish the incorporation of functional mature gp120/gp41 trimers into HIV-1 particles [31,32,33].

GBP5 is a member of IFN-inducible guanosine triphosphatases (GTPases) [34]. Members of the GBP family have been reported to antagonize a variety of invading pathogens including viruses, bacteria, and protozoa [35]. GBP1, a protein that is closely related to GBP5, inhibits a number of viruses including dengue virus, hepatitis C virus (HCV), encephalomyocarditis virus, and vesicular stomatitis virus (VSV) [36,37]. GBP5 was identified as a potential anti-HIV-1 factor in a genome-wide study for human genes sharing evolutionary signature of known restriction factors [38]. To supplement this identification, levels of GBP5 in primary CD4+ T cells and macrophages are enhanced by IFN-α, IFN-γ, IL-2, and TCR activation [31,34,39]. Not only does ectopic expression of GBP5 reduce the infectivity of HIV-1 particles by diminishing virion incorporation of gp120/gp41, depletion of GBP5 in primary macrophages elevates HIV-1 infectivity by enhancing the incorporation of mature gp120/gp41 into virions [31]. This action of GBP5 appears specific to retroviruses, since the function of VSV glycoprotein was not affected. GBP5’s location is crucial to its inhibitory effect on HIV-1; the intact C-terminal domain responsible of localizing GBP5 in the Golgi apparatus is required in lieu of GTPase activity. Ectopically introduced GBP5 causes two defects to HIV-1 Env protein as detected with Western blotting; gp160 cleavage is impaired, and glycosylation of HIV-1 Env is altered. It is currently unclear whether this altered glycosylation has contributed to the impaired cleavage of gp160. Within the GBP family, this anti-HIV-1 function appears to be specific to GBP5, as GBP1 does not affect HIV-1 Env despite its trans-Golgi localization. HIV-1 has a “trade-off” mechanism to partially overcome GBP5 inhibition through shutting down Vpu expression. Since Vpu and Env are synthesized from a single bicistronic mRNA, shutting down Vpu expression increases Env expression, which confers partial resistance to GBP5 [40]. Interestingly, the Vpu mutation that causes a loss of Vpu expression was identified in macrophage-tropic HIV-1 and some brain-derived HIV-1 strains, indicating that HIV-1 might have been pressured to resist high levels of GBP5 in macrophages [31,41].

The 90K protein (also known as Mac-2BP or LGAL3SBP) is an IFN-inducible, secreted immunostimulatory glycoprotein; it belongs to the family of scavenger receptor cysteine-rich (SRCR) domain-containing proteins [42]. In response to IFN-α stimulation, levels of 90K increase in various T cell lines, primary CD4+ T cells, and primary macrophages. Elevated levels of 90K have been reported in HIV-1 infected patients; hence, it was proposed as a serological marker of disease progression to AIDS [43,44]. 90K is N-glycosylated in the ER and Golgi complex before entry to the secretory pathway, thereby sharing the same route of trafficking and modification with HIV-1 Env protein [45]. Ectopic expression of 90K causes an accumulation of gp160, concomitant reduction of gp120, and loss of mature gp120/gp41 in HIV-1 particles, which result in impaired infectivity of nascent HIV-1 particles [32]. In addition, knockdown of 90K with siRNA in primary macrophages increases virion-associated gp120 and HIV-1 infectivity. It is also noted that both GBP5 and 90K are highly expressed in macrophages and may contribute to the low infection of macrophages by HIV-1. 90K inhibits both R5 and X4 HIV viruses [32]. While 90K also affects the furin-dependent maturation of Ebola GP, it minimally changes the processing of influenza virus HA0 protein and cellular glypican-3, suggesting a selectivity of 90K action on furin substrates. Lastly, the inhibitory effect of 90K on HIV-1 Env might be indirect, given the lack of detectable interaction of 90K with HIV-1 Env [32].

The anti-HIV-1 activity of 90K has been further mapped to the two central protein-binding domains of BTB-POZ and IVR, whereas the N-terminal scavenger receptor cysteine rich (SRCR)-like domain is dispensable [32]. However, a mutagenesis study by a different group showed that one truncation mutant of 90K (1-95) inhibits Env processing, while another 90K mutant (124-585) inhibits virion production [46]. Studies by Wang and colleagues also showed that 90K inhibits HIV-1 virion production by interacting with Gag and vimentin (VIM) in trapping HIV-1 Gag to VIM filaments, suggesting an alternative anti-HIV-1 mechanism by 90K [46]. It is unknown whether HIV-1 has adopted any mechanisms to counter this factor. The antiviral function of 90K is conserved among primates except the rhesus macaque [47]. Interestingly, 90K impairs gp160 processing and reduces levels of gp120 on the plasma membrane in other species, including the rhesus macaque; however, these functions do not always reduce the infectivity of nascent HIV-1 virions [47]. Further studies have shown that the impairment of mature gp120 incorporation into virions might also contribute to the antiviral action of 90K [47].

IFITM3 also causes the accumulation of gp160 and loss of mature gp120/gp41 in HIV-1 particles [33]. IFITM3 is a member of the IFITM family, including IFITM1, IFITM2, IFITM3, IFITM5, and IFITM10. Among these, IFITM1, 2, and 3 are interferon-inducible and have been shown to inhibit a wide range of viruses (Reviewed in [48]). Ectopic expression of IFITM3, and to a lesser extent IFITM2, impairs gp160 cleavage, promotes gp120 shedding, and diminishes the level of mature gp120/gp41 in HIV-1 particles, thus reducing HIV-1 infectivity [33,49]. In departure from GBP5 and 90K, IFITM3 has been shown to associate with both gp160 and gp120/gp41, which may allow IFITM3 to directly interfere with processing of the gp160 precursor. 

### 3.3. MARCH1, MARCH2, and MARCH8: Removing HIV-1 Env from the Cell Surface

Reaching the plasma membrane does not warrant safety for Env. MARCH8 (membrane-associated RING-CH 8) has been recently reported to modify HIV-1 Env and envelope proteins of other viruses to further downregulate them from the cell surface. MARCH8 is one of the 11 members of the MARCH family of RING-finger E3 ubiquitin ligases. As a transmembrane protein, MARCH8 bears a C4HC3 RING finger domain in the N-terminal cytoplasmic tail that recruits the E2 enzyme [50,51]. MARCH8 is involved in downregulating multiple transmembrane proteins, including but not limited to MHC-II [52], TRAIL receptor 1, and transferrin receptor [53]. Association of MARCH8 with these cellular transmembrane proteins often causes polyubiquitination of the target protein, followed by its trafficking to lysosomes for degradation. Another recently reported substrate for MARCH8 is BST-2, and MARCH8 regulates its ubiquitination, trafficking, and turnover [54]. Given its prolific regulation of cellular transmembrane proteins, it is thus not surprising that MARCH8 also targets viral envelope proteins and downregulates them from the cell surface [55].

When ectopically expressed, MARCH8 antagonizes not only HIV-1 Env but also glycoproteins of HIV-2, SIV, MLV, xenotropic MLV-related virus (XMRV), and VSV, suggesting a broad antiviral function. Mutating the RING domain, such as CS and W114A mutations, abrogates the antiviral activity of MARCH8, which demonstrates its dependence on E3 ligase activity. It is important to note that MARCH8 may impair different viral glycoproteins by different mechanisms. For example, MARCH8 removes HIV-1 Env from the cell surface, which is then retained within lysosomes without degradation. As a result, the total level of Env protein in cells does not change. In contrast, VSV G protein is downregulated by MARCH8 both at the cell surface and within the cell due to its degradation in lysosomes. Regardless of mechanistic details, MARCH8 interacts with both HIV-1 Env and VSV G proteins and likely alters their levels through ubiquitination. Further examination of antiviral activity exhibited by other members of the MARCH family revealed that MARCH1 and MARCH2 have antiviral functions similar to those observed in MARCH8; these members of MARCH family inhibit HIV-1 infectivity by downregulating HIV-1 Env from the cellular surface and reducing the levels of Env incorporated into the virions [56,57]. Similar to MARCH8, MARCH1 and MARCH2 are also localized at the plasma membrane [56]. As observed for MARCH8, MARCH1 also gets incorporated into virions; however, virion incorporation of MARCH2 remains controversial [56]. One group showed that HIV-1 infection increased MARHC2 expression but MARCH2 was not detected in the released virus particles [57], whereas another group showed that, similar to MARCH1 and MARCH8, MARCH2 is also found in progeny virions [56].

Higher levels of MARCH1, MARCH2, and MARCH8 were detected in myeloid cells such as monocyte-derived macrophages (MDM) and monocyte-derived dendritic cells (MDDCs) in comparison to primary CD4+ T cells. Unlike MARCH8, expression of MARCH1 and MARHC2 is highly inducible by type I IFN in MDM and MDDCs [55,56]. Knockdown or knockout of MARCH8 in myeloid cells increases HIV-1 infectivity, suggesting MARCH8 as one of the cellular factors that restrains HIV-1 infection of macrophages and dendritic cells. HIV-1 Vpr, Nef, and Vpu do not antagonize MARCH proteins; as a result, it remains to be determined how HIV-1 and other viruses, especially those that replicate in macrophages and dendritic cells, evade inhibition by MARCH1, MARCH2, and MARCH8. Since these MARCH proteins remove HIV-1 Env from the plasma membrane, it is speculated that HIV-1 takes advantage of these proteins to escape immunosurveillance.

## 4. Cellular Antagonists of Env Protein in HIV-1 Particles

In addition to targeting Env in the infected cells and preventing its incorporation into virus particles, some cellular factors such as IFITM3 and SERINC5 also find their way into virus particles and block the fusion of viral membrane and cellular membrane.

Beyond its impairment of gp160 processing in HIV-1 producing cells, IFITM3 is incorporated into HIV-1 particles. Virion incorporation of IFITM3 is at least partially due to its interaction with HIV-1 Env protein, as shown by co-immunoprecipitation [33,58]. Compared to IFITM3, IFITM2 demonstrates weaker inhibitory activity, whereas IFITM1 shows the least anti-HIV-1 activity [33,49,59]. One mechanism behind this impairment of HIV-1 infectivity is the reduction of gp120 in HIV-1 particles when HIV-1 is produced from 293T cells transfected with IFITM3 DNA and proviral DNA [33]. Alternative mechanisms may also exist, since IFITM3-bearing HIV-1 particles produced from CD4+ U87 cells are also less infectious but without detectable defects in viral Env protein [60]. In addition to HIV-1, IFITM proteins have also been detected in the particles of a large group of enveloped viruses, namely, murine leukemia virus (MLV), Mason–Pfizer monkey virus (MPMV), VSV, measles virus (MeV), Ebola virus (EBOV), West Nile virus (WNV), dengue virus (DENV), and Epstein–Barr virus (EBV), all leading to a decreased viral infectivity [61]. IFITM proteins’ broad spectrum of antiviral activity suggests a general mechanism that recruits IFITM proteins into different viruses to dampen viral infectivity. 

Another factor in virions, SERINC5, is a member of the serine incorporator (SERINC) family. As its name indicates, SERINC proteins are involved in the synthesis of two serine-containing lipids: phosphatidylserine and sphingolipids [62]. In 2015, two groups reported that in the absence of HIV-1 Nef protein, SERINC5, and to a lesser extent SERINC3, is incorporated into HIV-1 particles and impairs HIV-1 infectivity [63,64]. SERINC5 contains 11 transmembrane domains, and is associated with lipid rafts where HIV-1 particles often form. Presence of SERINC5 in HIV-1 particles obstructs the formation of the viral fusion pore [65], thus inhibiting HIV-1 entry into target cells. This mechanism of action likely results from an increased rigidity of viral membrane that bears clustered SERINC5, rather than altered lipid composition of viral membrane by SERINC5 [66]. SERINC5 interferes with the conformation of the MPER region of Env, which may contribute to its inhibition of Env-mediated cellular entry [65,67]. HIV-1 and other viruses have evolved countermeasures to antagonize SERINC5. HIV-1 Nef protein downregulates SERINC5 from the plasma membrane via the endosome/lysosome pathway, thus preventing SERINC5 incorporation into HIV-1 particles [68]. Interestingly, the Env protein of some HIV-1 strains are refractory to SERINC5 inhibition, which is analogous to the SERINC5-resistant property of VSV G, Ebola GP, and other viral envelope proteins [64]. In addition to these viral antagonists, the glycoGag protein of gammaretroviruses (such as MLV) and the S2 protein of equine infectious anemia virus (EIAV) have also been reported to overcome the antiviral function of SERINC5 [69,70]. The anti-SERINC5 strategies from different viruses indicate its broad antiviral function.

## 5. Env Antagonists in the Membrane of Target Cells: The Other Half of the Fusion Story

HIV-1 entry is marked by the fusion of viral and target cell membranes; this process is driven by the sequential conformational changes of Env trimer as a result of binding to receptor CD4 and co-receptor CCR5 or CXCR4. In addition to the series of host inhibitory mechanisms discussed above present in virus producing cells that target and disable Env in the viral membrane, target cell membrane is also equipped with mechanisms to prevent fusion with viral membrane. One prominent example is the antiviral function of IFITM proteins, which was discovered in a genome-wide siRNA screen for host factors that modulate the infection of influenza A virus [71]. Subsequently, IFITM proteins were shown to inhibit HIV-1 entry in a shRNA-based screen aiming to identify anti-HIV-1 ISGs [14]. Mechanistic studies further revealed that these IFITM proteins hamper viral membrane hemi-fusion and/or block the formation of fusion pore in virus target cells, due to the increased rigidity and altered curvature of IFITM-bearing cellular membranes [72,73]. IFITM proteins may modulate membrane fluidity by interfering with intracellular cholesterol homeostasis [72,73,74,75], although the involvement of cholesterol is still controversial [75]. Entry deterrence of incoming viruses also benefits from the subcellular localization of IFITM proteins at the plasma membrane and in endosomes/lysosomes, which covers the route of virus entry [76,77,78]. The N-terminal sequences of IFITM2 and IFITM3 bear the YMEL motif that binds to AP-2, and guides its endosomal and lysosomal localization via the endocytic pathway [78]. In contrast, IFITM1 lacks this endocytic motif; rather, it carries a KR-sorting motif at the C-terminus, which guides IFITM1 trafficking to recycling/early endosomes [79,80].

IFITM proteins are not the sole defense in target cells against virus entry. Cholesterol-25-Hydroxylase (CH25H), another ISG, protects target cells from viral infection [81]. CH25H produces a soluble oxysterol, 25-hydroxycholesterol (25-HC), which inhibits a large group of viruses including murine herpesvirus 68 (MHV68), VSV, Zika virus, HIV-1, herpes simplex virus 1 (HSV-1), EBOV, Nipah virus, Russian Spring–summer encephalitis virus, Rift Valley fever virus, and hepatitis C virus [82,83,84,85]. In addition to protecting its producer cells, 25-HC can be secreted to restrict virus entry into surrounding uninfected cells [83]. While 25-HC has been reported to regulate cholesterol biosynthesis and maintain cholesterol homeostasis, this function has been challenged by the observation that CH25H-deficient mice demonstrated normal cholesterol metabolism [86,87,88]. Clinical evidence from patients suffering from a hereditary disease, spastic paresis, that displays a high level of 25-HC but a normal level of cholesterol further disputes the role of 25-HC in cholesterol regulation. In contrast, accumulating evidence suggests an upregulation of CH25H in macrophages and dendritic cells upon inflammatory stimulation [81,89,90]. Furthermore, it was reported that accumulation of 25-HC instead of cholesterol in the lipid membrane prevents HIV-1 Env-mediated membrane fusion by modifying the secondary structure of the HIV-fusion peptide [91]. Analogous to IFITM proteins, 25-HC also operates in virus-producing cells by altering the glycosylation of Lassa virus glycoprotein [92]. Interestingly, 25-HC does not spare non-enveloped viruses, unlike IFITM proteins [93]. For example, 25-HC was shown to hamper reovirus uncoating [93]. Again, IFITM3 and 25-HC are thematically resonant in their antiviral actions, given the observation that IFITM3 likely prevents reovirus entry by delaying the proteolytic processing of reovirus particles within late endosomes [94]. Independent to producing 25-HC, CH25H also operates by directly acting on viral proteins. For example, the catalytically inactive CH25H mutant retains its antiviral function against HCV and porcine reproductive and respiratory syndrome virus (PRRSV) through direct interactions with NS5A of HCV and nsp1α of PRRSV [84]. 

## 6. Env Protein Fights Back: Evasion of Host Restriction on Virus Entry and Beyond

In order to replicate and transmit, viruses need to counter and evade the multi-layered host restriction defense system. Identification of viral antagonism against a host restriction factor also demonstrates the presence of this restriction in the context of in vivo viral infections, which have driven the selection and evolution of specific viral counter measures. Indeed, viral antagonistic strategies have been discovered for some host restriction mechanisms targeting HIV-1 entry, which began to illuminate the diversity of viral evolution in evading host restriction.

One viral countermeasure is to use a viral protein to target and downregulate the host restriction factor, which is well illustrated by the downregulation of SERINC5 by HIV-1 Nef, MLV glycoGag, and EIAV S2 proteins. A second strategy is to counter restriction factor inhibition through an indirect escape mechanism. One example of this mechanism is to increase the level of Env expression to counter host inhibition of virus entry, exemplified by HIV-1 escape from GBP5 inhibition through shutting down Vpu to elevate Env expression. Another example of viral adaptation and escape has been shown through Vif-null HIV-1 viruses conferring full resistance to APOBEC3G (A3G), which has been linked to a novel Env-dependent mechanism [95]. Env adaptation in Vif-null HIV-1 virus decreases virus fusogenicity and leads to higher levels of Gag-pol packaging into virions, which increases the levels of reverse transcriptase (RT). This Env-mediated elevation in RT levels prevents A3G-mediated hypermutation [95].

The third known strategy is to change Env protein sequence and thus adjust its entry function to gain resistance to host restriction of virus entry. We observed this viral escape mechanism when passaging HIV-1 in IFITM1-expressing SupT1 cells with the goal to select IFITM1-resistant viruses. The resistance mutations that enhanced HIV-1 cell-to-cell transmission were identified in viral Vpu and Env proteins, and they rescued HIV-1 replication in IFITM1-expression SupT1 cells [96]. Similarly, we identified Env mutations that enhance HIV-1 replication in IFITM3-expresing cells [33]. We later found that changing the V3 loop alone in Env can confer resistance to IFITM3 inhibition [49]. The ability of HIV-1 Env protein to resist IFITM3 was also observed in the transmitted founder HIV-1 strains [60]. This study convincingly showed that Env mutations, which arise to evade autologous antibodies 6 months after infection, transform the IFITM3-resistant HIV-1 to an IFITM3-sensitive one. Molecular and structural features of HIV-1 Env that determine its susceptibility and resistance to IFITM3 inhibition remain to be fully elucidated. A related envelope protein-mediated evasion was reported for influenza A virus [97]. It is known that IAV tends to finalize membrane fusion at a low pH in late endosomes/lysosomes, where IFITM3 is abundantly present. To escape from IFITM3 restriction, IAV HA protein can adapt to mediate membrane fusion in early endosomes where pH is relatively less acidic and has low levels of IFITM3 [97].

It appears that viral envelope protein resists more than just IFITM proteins. We and others have found that Env proteins of some HIV-1 strains, including transmitted founder HIV-1 isolates, are able to resist SERINC5 inhibition even in the absence of viral Nef protein which acts as a SERINC5 antagonist [64,67]. We further mapped the resistant determinant to the V3 loop of Env. Viral envelope has also demonstrated the capacity of overcoming host restrictions beyond virus entry. For example, passage of SIV/HIV chimeric virus (SHIV) in macaques in the presence of interferon-α led to the selection of interferon-α-resistant SHIV [98]. This resistance phenotype was mapped to viral Env protein that had a higher level of expression from the resistant virus. A separate study reported resistance of transmitted founder HIV-1 to type II interferon (interferon-γ), and this resistance activity was also mapped to viral Env [99]. In support of Env’s role in countering interferon-γ, replication of the sensitive HIV-1 strain in the presence of interferon-γ selected for resistance mutations in viral Env protein [99]. These studies report a general role of HIV-1 Env protein in generating resistance to interferon suppression. One possibility is that HIV-1 changes Env to acquire higher replication capacity in compensating for the loss of infectivity as a result of interferon inhibition.

In addition to this compensatory mechanism, viral envelope proteins are able to directly counter specific host restriction factors. One example is the antagonization of tetherin by HIV-2 Env, HERV-K Env, and Ebola glycoprotein [100,101,102]. Tetherin is known to inhibit the release of HIV-1 and many other enveloped viruses by tethering the progeny virions to the cell surface [103,104]. HIV-1 uses Vpu to nullify tetherin, while some primate lentiviruses such as SIVs from chimpanzee, sooty mangabeys, and African green monkeys use Nef to antagonize tetherin [105]. In contrast, HIV-2 and some SIV lineages including SIV tetanus do not encode for Vpu, they instead use Env as a tetherin antagonist [100,106]. Functional domain analysis revealed that Env’s antagonist activity against tetherin depends on a tyrosine based motif (YXX∅) in the cytoplasmic tail of the gp41 membrane proximal region [100]. Upon direct interaction between the extracellular domain of Env and the ectodomain of tetherin, the tyrosine motif (YXX∅) of Env gp41 recruits AP-2 complex and induces intracellular sequestration of tetherin from the cell surface and its accumulation in the trans-Golgi network [100,107,108]. Recent studies on HIV-2 isolates from different patients reveal that the anti-tetherin activity is a conserved function of HIV-2 Env [109]. Some ancient retroviruses might have also used their Env proteins to overcome tetherin inhibition, since the youngest and most active endogenous retrovirus (ERV) in human genomes, HERV-K, still preserves this function through its Env protein [101]. In addition to retroviruses, the glycoproteins (GPs) of Ebola virus and Lluvia virus are also antagonists of tetherin. Ebola GP does not remove tetherin from the cell surface but appears to depend on a GxxxA motif in its transmembrane domain [110].

## 7. Viral Envelope Protein under the Suppressive Pressure of Both Adaptive and Innate Immunity

Adaptive and innate immunity cooperate to create a higher genetic barrier for viral envelope protein to escape compared to either immune response alone. For example, HIV-1 Env is engaged in a constant battle with the antibody-mediated adaptive immune response. The ability of Env to evade an innate immunity might be limited by the need to resist antibody attack. This scenario is illustrated by the loss of IFITM3 resistance in transmitted founder HIV-1 strains, which mutate Env in order to resist autologous antibodies as infection progresses [60]. One implication of this finding is that relatively high level of IFITM3 may mount high enough inhibitory pressure to limit Env mutation pathways in evading inhibition by antibodies, thus creating a synergistic control of HIV-1 infection. It is equally possible that given the interferon-inducible nature of IFITM3 expression, subsidence of interferon response after the acute stage of HIV-1 infection may lead to reduction in IFITM3 level, thus mounting less pressure on HIV-1 Env and allowing Env to change and resist neutralizing antibodies.

The potential interplay of adaptive and innate immunity may also explain the need for Nef to antagonize SERINC5, even though HIV-1 Env has full capacity of SERINC5 resistance. This requirement is likely because certain types of neutralizing antibodies, such as those targeting the MPER sequence of Env, are able to sensitize the otherwise resistant Env to SERINC5 inhibition. This example demonstrates that dual pressures from SERINC5 (innate immunity) and neutralizing antibodies (adaptive immunity) have driven HIV-1 to evolve Nef’s antagonism against SERINC5. Further research could illuminate the synergistic suppression on viral envelope protein from both adaptive and innate immunity, and the seemingly endless evolution of viral counter measures to ensure viral survival.

## 8. Conclusions

A growing body of studies demonstrate that HIV-1 Env protein is not only the primary antigen of adaptive immunity but also the main target of innate immunity. An arsenal of antiviral proteins have already been discovered that either limit the synthesis of Env protein, deregulate Env glycosylation, impair Env cleavage by furin, or impede the incorporation of mature Env trimers into HIV-1 particles. In addition, some antiviral factors, such as SERINC5, IFITM3, and 25-HC restrict HIV-1 entry not by acting on Env directly but by altering the physical property of viral membranes or cellular membranes, which often enable them to inhibit a broad range of viruses. Cells use these diverse molecular mechanisms to inhibit the entry of many viruses far beyond HIV-1, which illustrates that targeting virus entry is a general and important host antiviral strategy (Figure 1, Table 1). Future research is expected to provide further insights into the molecular mechanisms by which each of these antiviral proteins restricts virus entry, to elucidate how these factors function together in vivo to create an optimal antiviral effect, and to understand viral countermeasures and escape mechanisms. It will also be interesting to investigate how these antiviral proteins, through altering the Env glycoprotein, modulate adaptive immune responses. The bnAbs, key effectors in adaptive immunity, are now being tested in clinical trials as a new HIV treatment [111]. Likewise, restriction factors, forming a key layer of innate immunity, also promise new approaches to treat and even cure HIV infection, such as the application of TRIM5α in gene therapy to create HIV-resistant hematopoietic stem cells [112,113].

## Figures and Tables

**Table 1:** Summary of the restriction factors that target HIV-1 Env.

Restriction Factor	Impact on HIV-1 Env	Other Enveloped Viruses Affected	Virus Escape Mechanism	References
ErManI	Decrease Env expression via ERAD pathway; modulate glycosylation of HIV-1 Env	IAV	HIV Vpr increases Env expression	[24,26,27]
GBP5	Impair cleavage of gp160; alter glycolysation of HIV-1 Env	MLV	Viral trade-off mechanism to increase Env expression by shutting down Vpu expression	[31,38]
90K	Prevent gp160 processing; decrease mature gp120/gp41 in virions	EBOV	TBD *	[32,47]
IFITM2/3	Deter viral entry into virus target cells; impair gp160 processing; promote gp120 shedding; decrease mature gp120/gp41 in virions; incorporate into virions and impair viral entry;	MLV, WNV, MPMV, EBOV, EBV, MeV, DENV	Overcome by HIV-1 Env	[33,61]
MARCH1/2/8	Downregulate Env from the plasma membrane	HIV-2, SIV, MLV, VSV	TBD	[55,56,57]
SERINC5	Impair virus infectivity; incorporate into virus particles; affect the conformation of the MPER region of Env	MLV, EIAV, EBOV	Downregulated by Nef from plasma membrane; countered by HIV-1 Env	[63,64,65,67]
25-HC	Modify the secondary structure of the HIV-fusion peptide; prevents membrane fusion	VSV, ZIKV, EBOV, NiV, HCV, RVF	TBD	[83,84,85,91,92,93]

* TBD: To Be Determined.
